# Can Monkeys Make Investments Based on Maximized Pay-off?

**DOI:** 10.1371/journal.pone.0017801

**Published:** 2011-03-10

**Authors:** Sophie Steelandt, Valérie Dufour, Marie-Hélène Broihanne, Bernard Thierry

**Affiliations:** 1 Centre National de la Recherche Scientifique, Département Ecologie, Physiologie et Ethologie, Strasbourg, France; 2 Université de Strasbourg, Institut Pluridisciplinaire Hubert Curien, Strasbourg, France; 3 Scottish Primate Research Group, Centre for Social Learning and Cognitive Evolution, School of Psychology, University of St Andrews, Fife, United Kingdom; 4 Laboratoire de Recherche en Gestion et Économie, EM Strasbourg Business School, Université de Strasbourg, Strasbourg, France; Yale University, United States of America

## Abstract

Animals can maximize benefits but it is not known if they adjust their investment according to expected pay-offs. We investigated whether monkeys can use different investment strategies in an exchange task. We tested eight capuchin monkeys (*Cebus apella*) and thirteen macaques (*Macaca fascicularis*, *Macaca tonkeana*) in an experiment where they could adapt their investment to the food amounts proposed by two different experimenters. One, the doubling partner, returned a reward that was twice the amount given by the subject, whereas the other, the fixed partner, always returned a constant amount regardless of the amount given. To maximize pay-offs, subjects should invest a maximal amount with the first partner and a minimal amount with the second. When tested with the fixed partner only, one third of monkeys learned to remove a maximal amount of food for immediate consumption before investing a minimal one. With both partners, most subjects failed to maximize pay-offs by using different decision rules with each partner' quality. A single Tonkean macaque succeeded in investing a maximal amount to one experimenter and a minimal amount to the other. The fact that only one of over 21 subjects learned to maximize benefits in adapting investment according to experimenters' quality indicates that such a task is difficult for monkeys, albeit not impossible.

## Introduction

The foundations of decision research, and hence its contemporary shape, have been strongly influenced by thinking from disciplines like economics. Human investors adjust their decisions according to partners on the basis of expected pay-offs. They are supposed to make rational decisions and to revise their decisions in order to optimize satisfaction [1]. Animals can also maximize pay-offs. When individuals exploit an environment where resources are distributed in patches, they can leave the patch and search for a new one when the rate of pay-off falls below the average rate for the entire area [2], [3]. Rational strategies are then defined as those increasing fitness and are an outcome of natural selection [4]. The theory of biological markets in particular assumes that living beings can adjust their investment based on the offers potentially provided by several partners [5]. In non-human primates, individuals may vary their rates of grooming in exchange to access for commodities [6], [7]. They are able to invest, that is, to avoid immediately consuming some goods with the intent of winning more [8], [9]. We lack evidence, however, about their abilities to adjust quantitatively their investment to expected pay-offs.

Monkeys and great apes appear to possess many of the skills required to perform successful investments in various contexts. They can make inferences, categorize objects and understand tertiary relations [10]. They are also able to make ‘more’ and ‘less’ value judgments about discrete quantities [11]–[14]. Numerous studies showed that monkeys are good at recognizing magnitudes for values under 8. For instance, rhesus macaques reliably prefer the larger amount in choices of one versus two items, two versus three, and three versus four [15], [16]. Monkeys can also discriminate between larger numerical values when high ratios are involved [17]. Rhesus macaques can learn to select the stimulus with the larger number of dots when pairs of numerical values between 1 and 9 are presented [18], [19]. Similar results are found in squirrel monkeys and tufted capuchin monkeys with discrimination between discrete quantities of one to nine food items [20]–[24].

Non-human primates are also able to combine discrete quantities, which can allow them to adjust their investment quantitatively. When presented with two trays, each tray containing two separate sets of food items, chimpanzees and capuchin monkeys select the greater total, indicating that they consider the sum of items [25]–[27]. Both great apes and monkeys succeed in tasks where they have to choose between two covered sets of food items to which an experimenter visibly adds or removes items in unequal numbers (capuchin monkeys: [28]; chimpanzees: [13], [29], [30]; orangutans: [12]; rhesus macaques: [15]). Monkeys can differentiate between different contingencies in discrimination learning task where they have to distinguish between two cues to gain rewards [10]. They can also discriminate between experimenters who behave in different ways towards them. For instance, capuchins and macaques preferentially indicate a food location to the most cooperative partners [31], and they recognize those providing the higher pay-off [32]–[34]. Monkeys can thus use potential partners as a tool to gain more. On the other hand, while monkeys may instrumentalize conspecifics, they may limit this to anticipating their behavior and not their intentions. It should be emphasized that in most experiments they appear unable to recognize actions in term of goals contrary to great apes [35]–[37] (but see [33]).

With regard to future-oriented behaviors, several experiments show that apes and monkeys can accept to lose an immediate benefit to gain more later; they postpone gratification from some seconds to a few minutes in tasks where they are given a choice between an immediately available but less preferred reward, and a delayed but more preferred one [38]–[40]. They sustain similar delays of gratification when presented with food items accumulating at regular time intervals [41], [42]. Non-human primates also maximize their pay-offs in tests requiring them to exchange with an experimenter. Chimpanzees and capuchin monkeys can learn to attribute values to non-edible tokens and exchange them for food [43]–[45]. However, this set up implies training monkeys to understand the value of the tokens. Monkeys and great apes can also give food items to receive a qualitatively more desirable one [8], [9], [46]. In that case, the value of the food is directly measured by consumption. In a study where capuchin monkeys were allowed to eat part of an item before returning it, individuals were seen to nibble most of a food item before attempting to exchange the remains for a larger reward with a human experimenter [8], [47]. Also, non-human primates can wait longer for a return if the expected quantity of food is larger [47], [48].

Decision-making in primates relies on skills requiring them to take into account several factors involving evaluation of discrete quantities, physical or temporal cost, and partner's reliability to maximize their pay-offs. In this study, we tested tufted capuchin monkeys, Tonkean macaques and long-tailed macaques in an exchange task where each subject initially received four food rewards that they could either consume or give back. To maximize the pay-off, subjects had to adapt the amount of food items they gave initially – the investment – to the food amounts to be returned by two different experimenters. We investigated whether the subjects could invest differentially depending on the experimenter' qualities in term of income. One experimenter gave back a reward twice the amount of the subjects' initial investment (doubling partner, providing 0, 2, 4, 6 or 8 rewards if subjects returned respectively 0, 1, 2, 3 or 4 rewards), whereas the other always gave back a constant amount regardless of the subjects' initial investment (fixed partner, always providing 8 rewards regardless of the amount initially returned). To maximize food income, subjects had to respond in different ways to each experimenter, offering a maximal amount to the first one and a minimal amount to the second (Table 1).

**Table 1:** Number of rewards obtained from both experimenters and subjects' net income according to the number of raisins returned by subjects.

	Doubling partner		Fixed partner	
Returned number of raisins	Reward	Net income	Reward	Net income
0	0	4	0	4
1	2	5	8	11
2	4	6	8	10
3	6	7	8	9
4	8	8	8	8

Within one session, the subjects' net income, i.e. the amount of raisins non-invested by the subject plus those received after return. The subject maximises its gain by giving more (4 raisins, net income 8) to the doubling partner, and less to the fixed one (1 raisin, net income 11).

## Results

When giving less than four raisins to experimenters, subjects exhibited different ways to remove raisins from the initial amount. They either ate some and returned the remaining ones (Pis, Arn, Lad, Pao), put all of them in their mouth and spat some back (Sha, Rav, Lad, Syb, Sam), or shared the four raisins between both hands keeping the content of one and returning the content of the other (Kin, Sad, Syb). Each subject consistently used the same way across the different phases of study (except for Lad and Syb who alternated their removal procedures; they mainly used the second procedure but sometimes used the first one for Lad, or the third one for Syb).

### Phase 1

**Figure 1:**
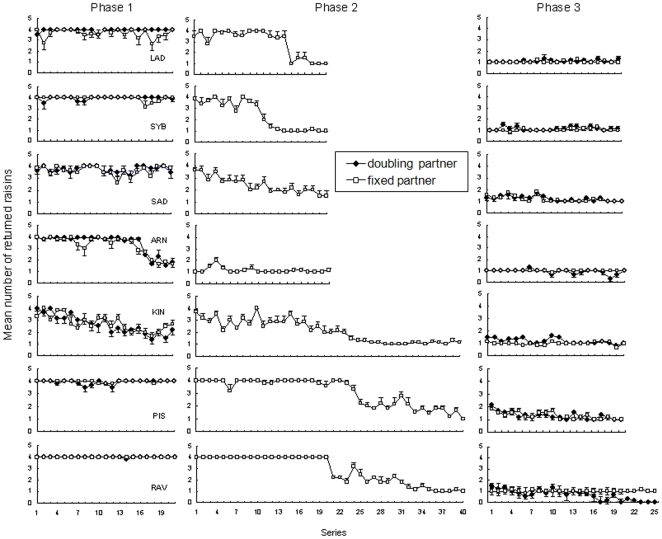
Number of raisins returned by seven subjects in Phases 1, 2 and 3. In Phases 1 and 3, subjects were tested with both doubling and fixed partners. In Phase 2, subjects were tested with the fixed partner only. Six subjects successfully modified their strategy in Phase 2 except for Arn who already changed of behavior at the end of Phase 1. In Phase 3, Rav returned 1 raisin then stopped exchanging with the doubling partner. Each plot represents the mean number of raisins returned in one session of six trials, along with standard errors.

In this phase, 20 subjects failed to adapt the amount of given raisins according to partners' quality during 21 sets of two sessions (Figure 1 and S1). Among subjects, seventeen consistently gave all four raisins to the doubling and fixed partners. Two other subjects (Sam, Pao) gave 1–3 raisins to both partners. A third subject (Arn) initially gave all four raisins, but after the 16^th^ set of two sessions, he learned to give 1–2 raisins to both partners. Comparing the performances of subjects according to partners' quality in the last 10 sets of sessions did not yield significant differences (fixed partner: mean number of raisins ± sd  = 3.55±0.49, doubling partner: m = 3.56±0.36, n = 20 subjects, T = 53.0, p = 0.642).

**Figure 2:**
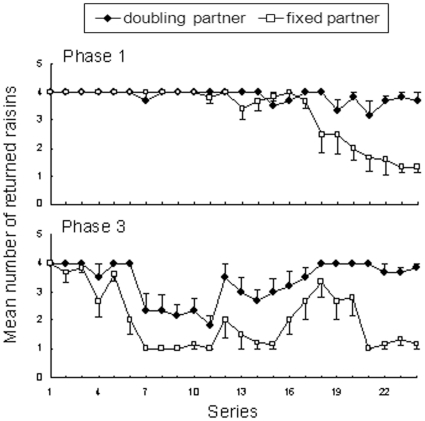
Number of raisins returned by the subject Sha in Phases 1 and 3.

One subject was able to adjust his behavior according to experimenters' quality. This Tonkean macaque (Sha) was tested during 24 sets of two sessions. From the 17^th^ set, he gave a decreasing numbers of raisins (three to one) to the fixed partner while consistently returning 3–4 raisins to the doubling partner (Figure 2). Comparing his performances according to partners' quality during the last 10 sets of sessions yielded a statistically significant difference (fixed: m = 2.51±1.35, doubling: m = 3.60±0.91, n = 10 sets, T = 4.0, p = 0.016).

### Phase 2

In Phase 2, the 20 subjects that had previously failed to differentiate between partners' quality were tested in sessions involving a single fixed partner. Phase 2 was run to counterbalance the tendency of most subjects to return all 4 raisins in Phase 1. Among the 20 subjects, 13 maintained the main strategy used in Phase 1 (see Figure S1). The other seven subjects altered their behavior in the course of sessions. They learned to give 1–2 raisins to the fixed partner (Figure 1).

### Phase 3

The seven subjects who reduced the number of raisins they gave in Phase 2 were tested again in sessions involving two different experimenters (Figure 1). Among them, six continued to give 1–2 raisins to both partners as in Phase 2. Comparing the performances of subjects according to partners' quality did not yield significant differences (fixed: m = 1.05±0.10, doubling: m = 1.10±0.15, n = 6 subjects, T = 15.0, p = 0.144). A seventh subject (Rav) started to stop exchanging with the doubling partner, consuming the four raisins. Yet, he kept on giving one raisin to the fixed partner. The analysis showed that he responded differently to both partners' qualities (fixed: m = 1.02±0.13, doubling: m = 0.17±0.39, n = 10 sets, T = 55.0, p = 0.005).

The subject Sha, having differentiated between partners' quality in Phase 1, was tested in phase 3 with two new experimenters in order to confirm his response (Figure 2). His behavior progressed during the sessions. At first, he gave about 1–2 raisins to the fixed partner while generally giving 2–3 raisins to the doubling one. After several sessions, he gave 2–3 raisins to the fixed partner and four to the doubling. In the last sessions, he gave a minimal number (one) to the fixed partner and a maximal number (four) to the other partner. Analyzing his performances showed that he adopted contrasting strategies according to partners' quality (fixed: m = 1.23±1.26, doubling: m = 3.63±0.72, n = 10 sets, T = 55.0, p = 0.005).

### Net incomes in Phases 1 and 3

By experimental design the subjects' net income should differ according to experimenters' quality. We checked that it was larger with the fixed than with the doubling partner in the last 10 sets of sessions in Phase 1 for Sha (fixed: m = 9.49±1.01, doubling: m = 7.60±0.34, n = 10 sets, T = 55.0, p = 0.005) and other subjects (fixed: m = 8.42±0.77, doubling: m = 7.61±0.84, n = 20 subjects, T = 41.0, p = 0.001), and also in Phase 3 for Sha (fixed: m = 10.17±0.87, doubling: m = 7.63±0.35, n = 10 sets, T = 55.0, p = 0.005), Rav (fixed: m = 10.98±0.13, doubling: m = 4.18±0.39, n = 10 sets, T = 55.0, p = 0.005) and other subjects (fixed: m = 10.95±0.20, doubling: m = 5.10±0.27, n = 6 subjects, T = 21.0, p = 0.028).

In Phase 1, Sha received a total of 2414 raisins (1046 raisins with the doubling partner; 1368 raisins with the fixed partner; difference: 322 raisins). For other subjects, the total mean of raisins was of 2187 (983 raisins with the doubling partner; 1204 raisins with the fixed partner; mean difference: 221 raisins). In Phase 3, Sha earned a total income of 2478 raisins (1059 raisins with the doubling partner; 1419 raisins with the fixed partner; difference: 360 raisins). Rav had a total income of 2071 raisins (435 raisins with the doubling partner; 1636 raisins with the fixed partner; difference: 1201 raisins). For other subjects, the total mean of raisins was of 1855 (584 raisins with the doubling partner; 1271 raisins with the fixed partner; mean difference: 687 raisins).

## Discussion

A single subject (Sha) could maximize pay-offs by following different rules according to experimenters' quality. Most capuchins and macaques were not able to adapt the invested amount of food items to the potential returns from each experimenter. In Phase 1, most individuals consistently gave a maximal amount to both. Such strategy maximized pay-off with the doubling partner, but was inappropriate with the fixed one. Fewer subjects showed the reverse response pattern, giving a minimal number of raisins by the end of this phase. This strategy maximized pay-offs with the fixed partner, but not with the doubling. In Phase 2, subjects had to exchange only with the fixed partner. One third of them succeeded in maximizing pay-off and learned to give a minimal amount. Among these seven subjects, only one (Rav) discriminated between partners' quality in Phase 3 but failed to understand the rule that would bring him optimal benefits with the doubling partner. The others subjects maintained the same strategy as in Phase 2 and did not adapt their investment strategy according to partners' quality.

It might be argued that the experimental set-up did not provide time enough for subjects to adjust their behavior, but the fact that Sha learned to modify his behavior after some trials weakens this interpretation. An alternative explanation is that most subjects may have been unable to differentiate between experimenters' quality according to the food amounts that they returned. However, this explanation is also unlikely since it is known that monkeys are able to discriminate two experimenters behaving differently [31]–[34]. Moreover, most subjects sometimes gave back a different number of raisins to experimenters, thus getting an opportunity to learn that experimenters did not respond in the same manner. It should be emphasized that the net income differed according to experimenters' quality, since no subjects always gave 0 or 4 raisins. In Phase 1, subjects experienced a difference of close to one raisin between experimenters; and in Phase 3, the subjects' net income with the fixed partner was more than twice than with the doubling partner. Still, they did not adjust their behavior according to the partner' quality. Moreover, former studies have shown that monkeys succeed in tasks requiring them to discriminate between quantities [11]–[24]. When required to trade tokens for rewards with two different experimenters, tufted capuchins were able to select the one providing the higher pay-off [32]. Here, subjects had to do more than just choosing between two options, they had to draw different decision rules from the contrasting conduct of two different human partners. From previous work on discrimination learning, we know that it is quite demanding for animals who learned in a training phase to select one cue in a two-choice discrimination task to learn, in a following reversal phase, that the second cue is then rewarded [10], [49]. Our experimental situation was even more challenging since it required subjects to respond in a different way at each partner's quality change. It is therefore not surprising that most subjects failed to regularly alternate their decision rule in this repeated conditional discrimination task.

In Phase 2, seven subjects – three macaques and four capuchin monkeys – sized the opportunity to remove some raisins from the initial amount in order to maximize pay-off. This corroborates results previously found in a study where capuchins were observed nibbling part of the initial item before returning it [8], [47]. Therefore, failure to learn to keep some of the food was not what hindered success in this experiment. The fact that only a third of the monkeys succeeded is not too surprising. Indeed, monkeys were rewarded regardless of the number of raisins invested - even no exchange whatsoever rewarded them with the 4 raisins they kept. Therefore, there was no negative reinforcement for giving one quantity or another. Although we aimed to test whether monkeys could learn to differentiate between two experimenters' qualities, we did not want to condition them to do so. In each phase, it was up to them to realize the differences in the rewards obtained according to the quality of the experimenter they were interacting with. Previous studies have shown that monkeys could recognize when experimenters subtracted several items from a given number of incentives [50], [51]. In the present study, some subjects consumed some of the raisins and gave the remaining to the experimenter, whereas others first gave some raisins and then ate the remaining ones. In both cases, subjects were able to remove 2–3 items from the total amount before giving 2-1 items to the experimenter. We propose that subjects' decisions rested on their ability to recognize magnitudes, albeit in an imprecise way [52], [53].

From the seven individuals who started giving back minimal amounts in Phase 2, one behaved differently with each partner's quality of Phase 3; while he consistently gave one raisin to the fixed partner, he eventually stopped exchanging with the doubling one. Thus, this capuchin monkey was able to maximize pay-offs with the fixed partner and could recognize that the doubling partner might respond in a less satisfactory way. Still, he failed to understand which rule would bring him optimal benefits with this second partner.

It must be emphasized that one Tonkean macaque succeeded in optimizing pay-offs with both experimenters. He followed different decision rules with each experimenter's quality in Phase 1, and did it again with two new experimenters in Phase 3. By the end of each phase, he invested a maximal amount with the doubling partner and he removed most items before investing with the fixed one. To our knowledge, this represents the only example of decision-making by drawing different rules based on combination of discrete quantities in monkeys, and maybe even in great apes. The fact that only one of over 21 subjects could maximize benefits in adapting investment according to experimenters' quality indicates that such a task is difficult for monkeys, albeit not impossible.

Cognitive limits can underpin the present results, but we cannot exclude that different factors related to the design of the task concurred to create additional difficulties. First, one may argue that in Phase 3, monkeys only gave a minimal amount because they had been trained to do so in Phase 2, and that training at this stage would have also included training with the doubling partner. It is likely that training with only the fixed partner influenced their response greatly. However, training in Phase 2 was carried out to counterbalance the very strong tendency of the subjects to systematically give four raisins in Phase 1. If we had exposed them to both contingencies again, this could have forbidden the outcome “give as little as possible”. Thus, in the actual set up Phase 3 was run with the knowledge that the seven subjects involved had all been capable of both responses, giving either a maximum (Phase 1) or a minimum (Phase 2) number of raisins. Second, albeit statistically significant, the weak difference of net income between different experimenter's qualities (1 raisin), experienced by individuals in Phase 1 could be insufficient for monkeys to detect that they were not maximising rewards. It is known that monkeys can distinguish between weak differences of items [15], [16]. Moreover, in Phase 3 this difference was two-fold between experimenters. Nevertheless, subjects did not adjust their return according to experimenters' qualities. Finally, it is possible that some individuals may require more exposure to each partner's quality in order to learn how to adjust their return. Whenever individuals showed unstable strategies in each phase, additional sessions were run to allow for such learning to occur. This however did not lead to successful learning. Still, sufficient learning time is probably a critical requirement for the adequate mastering of such complex cognitive decision-making by most subjects. In humans, being able to follow multiple directions or to switch between decision rules develops slowly during childhood [54], [55]. Providing that sufficient learning time is allowed, and that monkeys can pay attention to differences in partners' quality, maximizing pay-off using opposite decision rule is within the reach of these species. In the present experiments we reduced the complex interactions commonly addressed by behavioral biology and economics to a simple dyadic situation in which subjects interact with a human experimenter. This is a current procedure in experimental cognition. Further research should attend more specifically to those additional factors – whether ecological, social or cognitive – liable to facilitate such learning in non-human primates and other animals.

Trading with multiple partners following different rules is characteristic of human economics; individuals make decisions based on their expectations regarding partners' responses. Here, monkeys had to adjust the amount to be returned according to their expectations about the behavior of two different experimenters. Our results may have implications regarding how non-human primates manage their relationships with conspecifics. The ability to adapt pay-offs according to the gains potentially brought by each partner could be related to the ability of individuals to invest more in one mate or another [56]–[58]. Future studies should compare monkeys and great apes to investigate whether the development of such abilities would have preceded the rise of economical transactions in humans.

## Methods

### Ethics Statement

Animals were given *ad libitum* access to food and water. All procedures complied with the recommendations of the Weatherall report. The research was conducted under license 67–100 from the French Agricultural Department (Préfecture du Bas-Rhin).

### Subjects

The subjects were maintained at the Primatology Center of the Strasbourg University. Their age and sex are presented on Table 2. We tested eight tufted capuchins (*Cebus apella*) belonging to a group of 18 individuals housed in an indoor-outdoor enclosure composed of several compartments totaling 78 m^2^. Four Tonkean macaques (*Macaca tonkeana*) belonged to a group of seven individuals housed in an indoor-outdoor enclosure composed of several compartments totaling 35 m^2^. Two other Tonkean macaques belonged to a group of 16 individuals raised in a 1-acre wooded area including a shelter and a 40-m^2^ wire-mesh fenced enclosure used for experiments. Three long-tailed macaques (*Macaca fascicularis*) were housed together in an enclosure of 10 m^2^ composed of several compartments and located in an indoor room. Four other long-tailed macaques were individually housed in the same room in cages of 125×80×80 cm. Animals were fed with commercial monkey diet. They were never deprived of food.

**Table 2:** Subjects participating in the study.

Subjects	Age (yrs)	Sex	Rearing conditions
Tufted capuchins			
Kin	16	female	group-living, indoor-outdoor
Ali	9	female	group-living, indoor-outdoor
Pao	7	female	group-living, indoor-outdoor
Arn	10	male	group-living, indoor-outdoor
Pis	7	male	group-living, indoor-outdoor
Pop	7	male	group-living, indoor-outdoor
Rav	6	male	group-living, indoor-outdoor
Sam	5	male	group-living, indoor-outdoor
Tonkean macaques			
Syb	5	female	group-living, indoor-outdoor
Rim	6	male	group-living, indoor-outdoor
She	5	male	group-living, indoor-outdoor
Sim	5	male	group-living, indoor-outdoor
Lad	11	female	group-living, semifree-ranging
Sha	5	male	group-living, semifree-ranging
Long-tailed macaques			
Lou	11	male	group-living, indoor
Ram	16	male	group-living, indoor
Sad	12	male	group-living, indoor
Cas	12	male	separated, indoor
Don	16	male	separated, indoor
Jac	15	male	separated, indoor
Joe	11	male	separated, indoor

### Testing Procedure

Subjects had been trained to exchange food items with humans prior to experiments [8], [59]. Most subjects had been involved in a delay-of-gratification task where they had to keep a piece of biscuit in their hand for a given amount of time before returning it for a better or larger reward. All subjects succeeded in waiting for more than 10 seconds in this task. The present study, by comparison, was based on an immediate exchange and imposed a lower need for self-control in all subjects. They were also involved in daily training sessions over a 3-month period where they had to give several Zante raisins to obtain twice the number of raisins. Another experiment gave subjects some background in discriminating between values of 6 and 18 food items [60].

**Figure 3:**
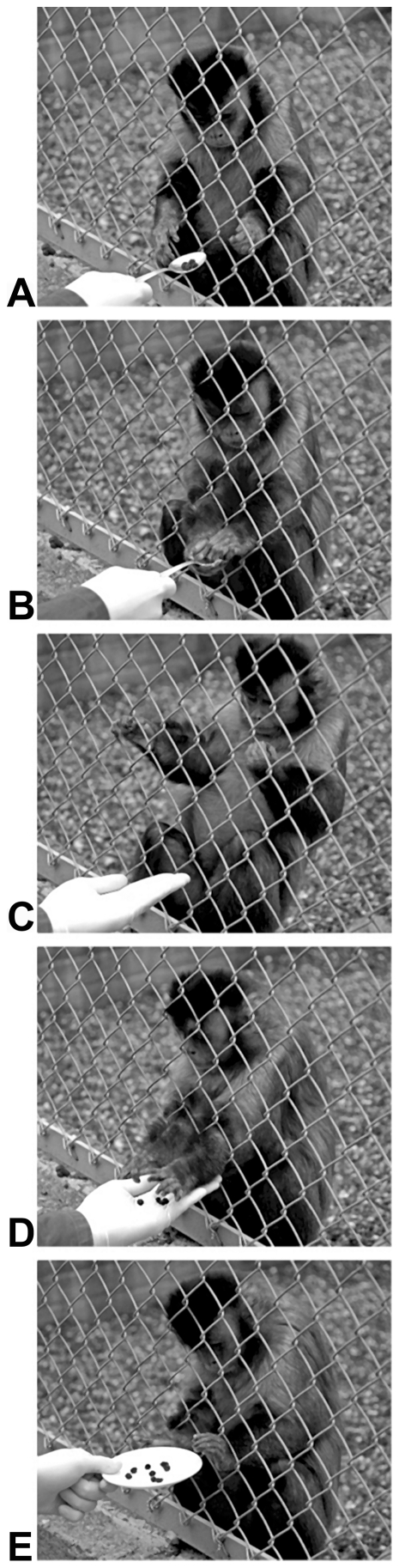
Exchange sequence between capuchin monkey and experimenter. (A) The experimenter presents four raisins on a spoon, (B) The subject is allowed to take the raisins, (C) The subject is requested to return the raisins, (D) The subject drops the raisins in the hand of the experimenter, (E) The subject receives eight raisins in a cup.

Group-living subjects were temporarily separated from their mates into individual compartments and later released back into their group. The experimenter sat in front of the wire mesh and laid four cups containing four potential rewards on the ground in full view of the subject. The number of potential rewards shown depended on the quality of the experimenter running the trial. A test started when the experimenter showed to the subject four raisins on a teaspoon for 2 s. Then she gave them to the subject. After 3 s, the experimenter held out a hand, palm open, in front of the subject requesting them back. When the subject gave one or more raisins, the experimenter rewarded the subject by supplying him/her with a corresponding, larger, number of raisins from one of the four potential cups (Figure 3). If the subject did not give raisins, the trial ended. We waited for 2 min after the end of food consumption before starting another trial.

### Experimental Design

Two different experimenters familiar to the subjects were involved in the testing phase. A first one, the doubling partner, always returned a number of raisins twice those given by the subjects. Therefore, potential rewards consisted in cups presenting either two, four, six or eight raisins. The second experimenter, the fixed partner, always returned eight raisins, regardless of the number of raisins given by subjects (one to four). Thus, potential reward consisted in one cup among four, each cup presenting eight raisins. The subjects' net income, i.e. the amount of raisins non-invested by the subject plus those received, could vary depending on which partner they interacted with (Table 1).

For training, a first 2 day-period was run where subjects were trained to give several raisins. Subjects were submitted to one daily session of six trials. A different experimenter from the two partners described above initially provided the subject with either one or four raisins and requested subjects to give them all to obtain eight raisins. Three trials were run in a random order for each condition. We did not require learning criteria for this step. In a second 2-day training period, subjects were familiarized to the doubling and fixed partners. They were exposed once (in a single trial) to the doubling partner and once to the fixed. In order for them to experience the difference in the reward amount, subjects had to give at least one raisin to each partner. If they failed, a second trial was run. Subjects needed between 2 and 4 trials to reach this criterion.

With regard to the testing phase, we first tested subjects in successive sets of two sessions (one session per partner) in a random order. There was no more than one session of six trials per half-day. The subjects' net income could vary within one session from 24 to 48 raisins with the doubling partner, and from 24 to 66 with the fixed partner. The partners' role differed and was counterbalanced across subjects; the doubling partner for 11 subjects was the fixed one for the remaining ten.

Because subjects failed to adapt their strategies according to the quality of the partner they were tested with, we ran them in a second phase involving the fixed partner only. We aimed to detect whether subjects could maximize their gain in a simplified version of the task. Phase 2 was run to counterbalance the tendency of subjects to return all 4 raisins in Phase 1. Indeed, during the training phase, all subjects had learned to return a maximum of raisins, which was the main behavior observed in Phase 1. In Phase 2 the goal was therefore to reinforce any subject who would start “giving less”. When subjects did choose the best strategy in the second phase (giving only one raisin to obtain eight ones), we tested them in a third phase, which replicated the procedure of Phase 1. Phase 3 was then run with the knowledge that the seven subjects involved had all been capable of both responses, giving either a maximum (Phase 1) or a minimum number of raisins (Phase 2). A single subject (Sha) directly passed from Phase 1 to Phase 3 because of success in Phase 1. Each phase involved different experimenters.

Whenever the strategy adopted by subjects was not stable at the end of each phase, and to ascertain that no learning trend was occurring, we added testing sessions until the performances' curve flattened. In Phase 1, subjects were tested in 21 sets of two sessions with the doubling and fixed partners; the first set was a learning period. One subject (Sha) was tested in 24 sets of two sessions. Phase 2 was composed of 20 sessions with the single fixed partner. Four subjects (Pao, Kin, Pis, Rav) were tested in 40 sessions. In Phase 3, subjects were tested in 20 sets of two sessions with both partners' qualities. We conducted 25 sets with Rav and 24 sets with Sha. Trials when subjects did not return any raisins (2.1% of trials) were discarded from data processing.

To test whether subjects responded differently to the fixed and doubling partners, we compared their performances at the individual or at the group level in the last part of each testing phase, i.e. the last 10 sets of sessions, using a Wilcoxon matched-pairs test (exact procedure, [61]) with SPSS software version 16 (SPSS Inc., Chicago IL, U.S.A.).

## Supporting Information

Figure S1 **Number of raisins returned by 13 subjects in Phases 1 and 2.** In Phase 1, each set is composed of one session with the doubling partner and another with the fixed one. In Phase 2, subjects were tested with the fixed partner only. They did not modify their strategy in this phase. Each plot represents the mean number of raisins returned in one session of six trials. Errors bars represent standard errors of the mean for each session. The subject Pao was tested for a larger number of sessions than others to ascertain that no learning trend occurred in its performances.
(PDF)
