# Supplementary figures and images for: Can Monkeys Make Investments Based on Maximized Pay-off?
Source: PLoS One. 2011 Mar 10;6(3):e17801. doi: 10.1371/journal.pone.0017801 (PMC3053400; doi:10.1371/journal.pone.0017801)

Figure S1. Number of raisins returned by 13 subjects in Phases 1 and 2.

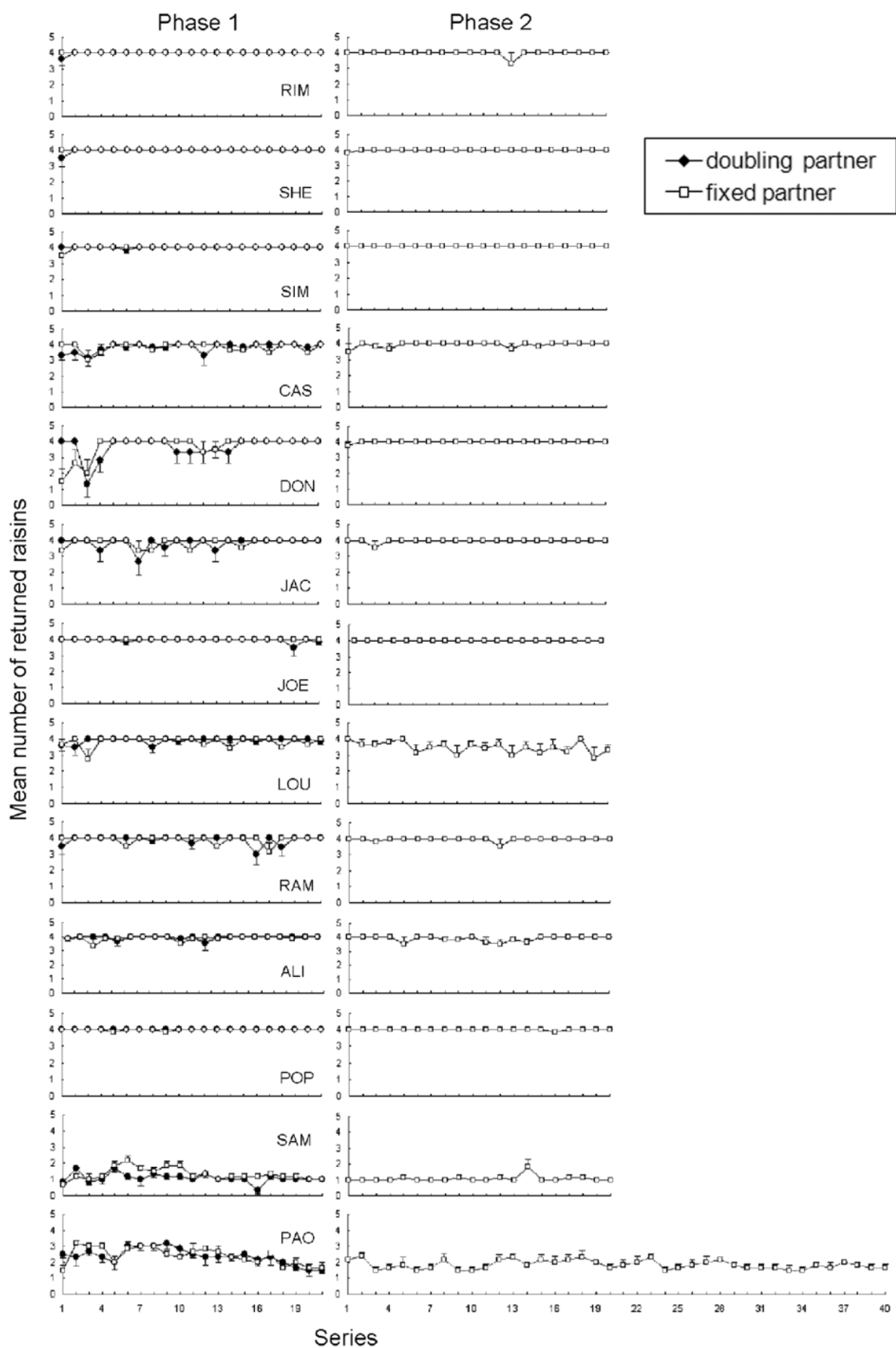

Supplement: Figure S1 — Number of raisins returned by 13 subjects in Phases 1 and 2. In Phase 1, each set is composed of one session with the doubling partner and another with the fixed one. In Phase 2, subjects were tested with the fixed partner only. They did not modify their strategy in this phase. Each plot represents the mean number of raisins returned in one session of six trials. Errors bars represent standard errors of the mean for each session. The subject Pao was tested for a larger number of sessions than others to ascertain that no learning trend occurred in its performances. (PDF) [file pone.0017801.s001.pdf]
